# Neurofibromatosis Type I and Stromal Tumor with a Multiple Digestive Localization

**DOI:** 10.1155/2021/2868966

**Published:** 2021-10-21

**Authors:** Amina Chaka, Farouk Ennaceur, Mohamed Amine Tormen, Ibtissem Korbi, Faouzi Noomen, Khadija Zouari

**Affiliations:** Department of General and Digestive Surgery at the University Hospital Fattouma Bourguiba of Monastir, University of Monastir, Tunisia

## Abstract

Neurofibromatosis type I (NF1) is also known as von Recklinghausen disease. It is a genetic disorder that affects the growth and development of nerve cell tissue, which is characterized by a multisystem disorder and an increased risk for cancer. The incidence of gastroduodenal stromal tumor during Recklinghausen disease can reach 35% in autopsies and 5% in clinical cases. In our case, the diagnosis of neurofibromatosis type I was made in a middle-aged women initially diagnosed with a pancreaticoduodenal tumor.

## 1. Introduction

Neurofibromatosis type I is also known as von Recklinghausen disease. It is a genetic disorder that affects the growth and development of nerve cell tissue, which is characterized by a multisystem disorder and an increased risk for cancer.

The incidence of gastroduodenal stromal tumor during Recklinghausen disease can reach to 35% in autopsies and to 5% in clinical cases.

In our case, the diagnosis of neurofibromatosis type I was made in a middle-aged woman initially diagnosed with a pancreaticoduodenal tumor.

## 2. Case Report

It is a 61-year-old woman, having a regular checkup for rheumatoid arthritis in the rheumatology department. She was admitted in our department for epigastralgia as well as pain on the right hypochondrium, evolving for two days without fever.

**Figure 1:**
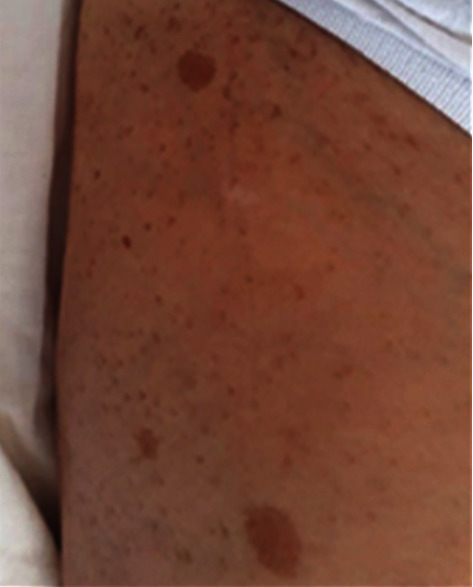
Café au lait spots or coffee with milk spots; it consists in hyperpigmented lesions that may vary in color from light brown to dark brown.

**Figure 2:**
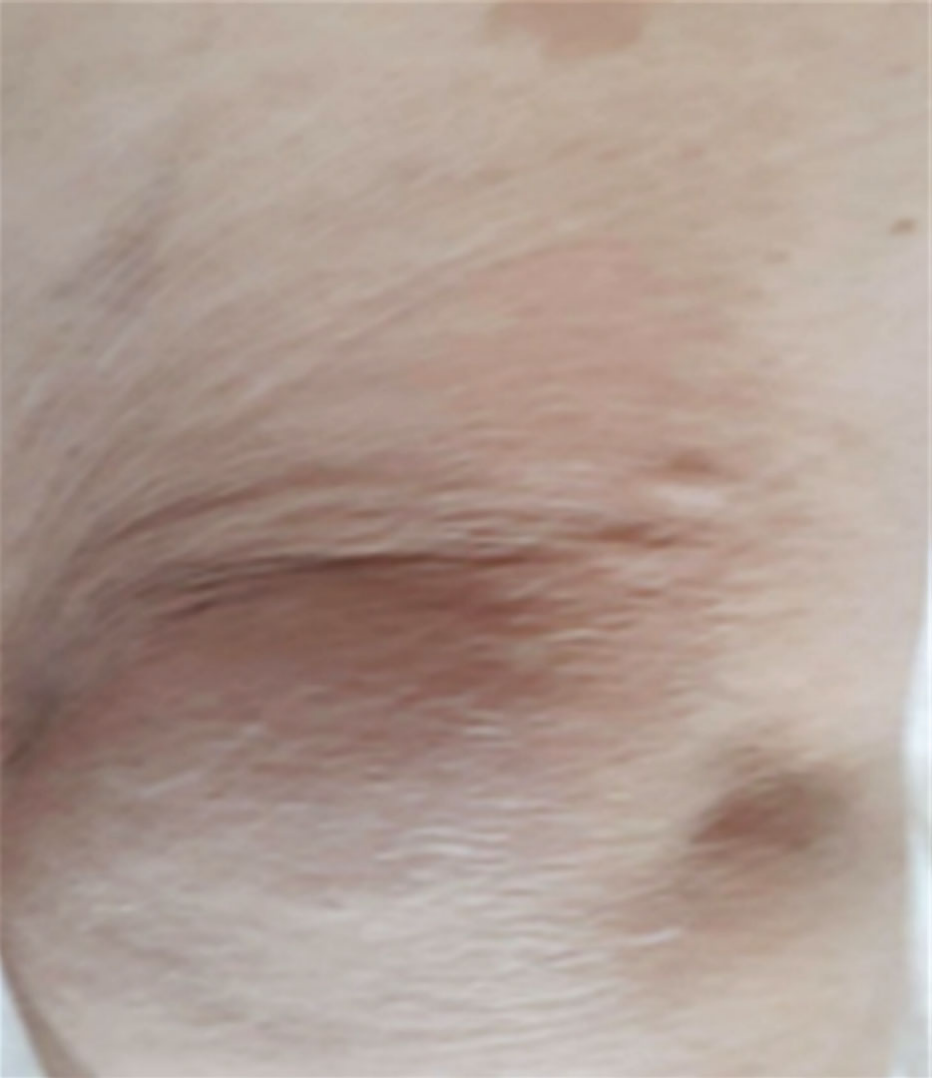
Neurofibromas on the anterior thigh.

The general examination of this patient did not show any anomalies. The patient was in a good general condition with a perfectly supple abdomen with no fever. However, we observed brownish skin lesions, also known as café au lait spots or coffee with milk spots that consists in hyperpigmented lesions that may vary in color from light brown to dark brown (Figure 1). Also, the patient has nodular cutaneous lesions with millimeter size and dots on the skin especially on the anterior surface of the thigh. These lesions correspond to neurofibromas (Figure 2).

**Figure 3:**
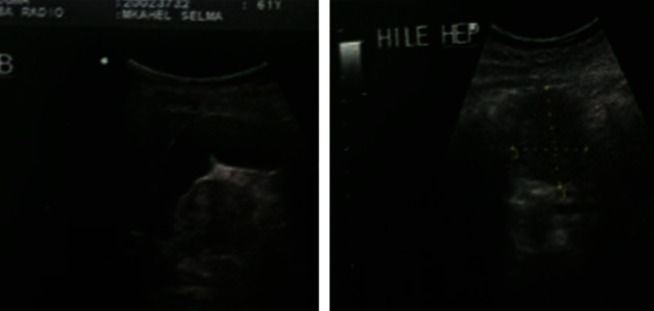
Abdominal ultrasound showing heterogeneous retroperitoneal nodular lesion, vascularized using color Doppler in presence of multiple partitions.

Abdominal ultrasound showed a heterogeneous retroperitoneal nodular lesion, vascularized with the presence of multiple partitions to the color Doppler (Figure 3).

**Figure 4:**
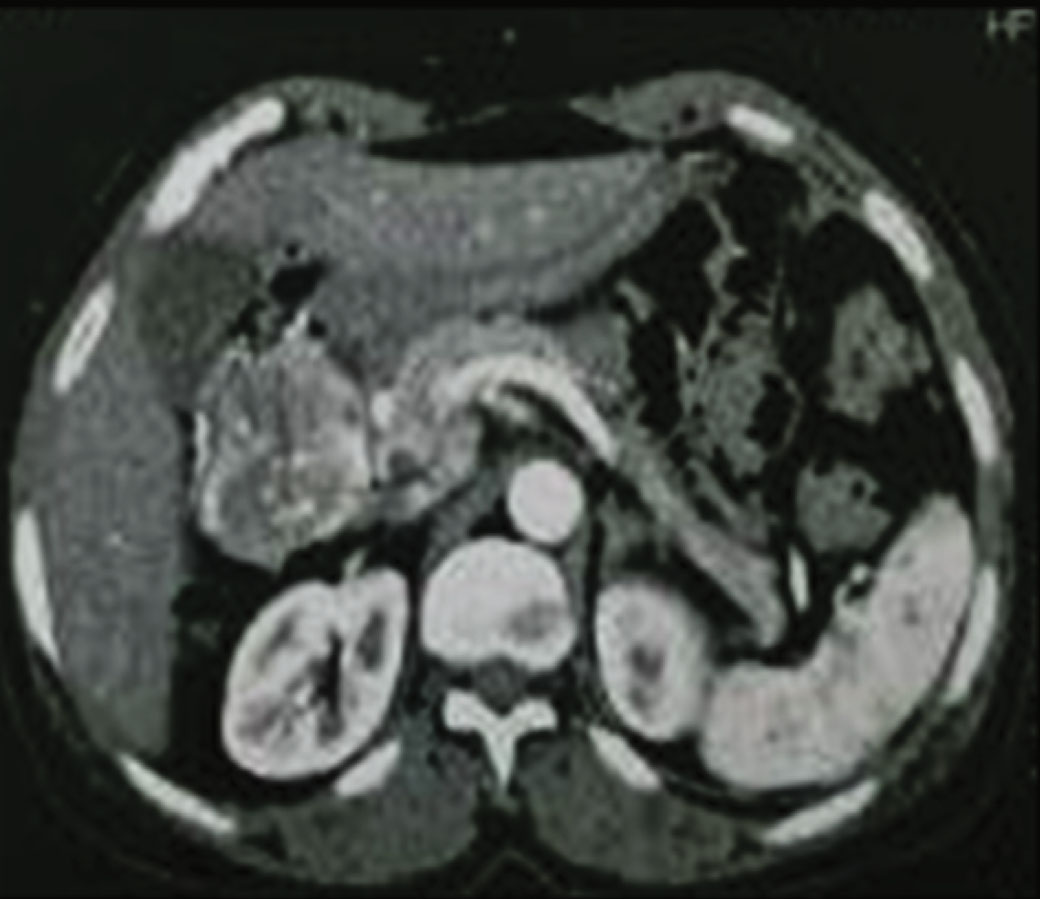
Abdominal CT enhanced scan shows a 5 cm exophytic duodenum mass, revealing a stromal tumor.

The level of tumor markers (CA19-9 and CEA) was normal. The abdominal CT scan showed a 5 cm juxta duodenal mass, suggesting a stromal tumor. It does not show any reverberation or signs of locoregional extension (Figure 4).

We completed our examination by performing a gastrointestinal endoscopy, which revealed a large, easily identifiable round and heterogeneous lesion which was calcified and vascularized, with collateral circulation growth and a tissue mass component of the pancreas measuring 5 cm. Immunohistochemistry analysis showed an elevated level of vimentin and alpha-fetoprotein (AFP) and the absence of keratin and Synaptophysin.

**Figure 5:**
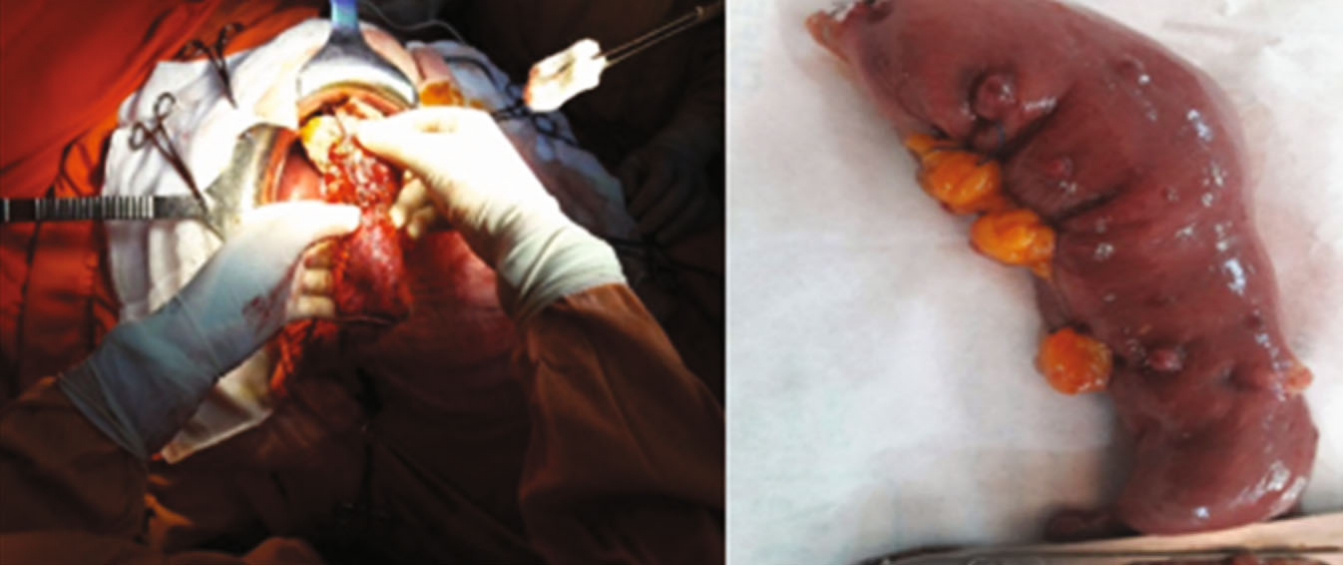
A tumor located 7 cm from the major axis, located at D1 with exophytic lesions, of centimeter size, located at the surface of the small bowel, resembling fibroids.

As a result, this patient was diagnosed with solid pseudopapillary tumor of the pancreatic cephalic area. The tumor extension assessment was negative, and an operability test was carried out. As a conclusion, the patient undertook a pancreaticoduodenectomy, and intraoperative exploration revealed a tumor measuring 7 cm, with a significant collateral venous circulation, located at D1 and independent of the pancreas (Figure 5). The exploration confirmed the existence of multiple small exophytic lesions, located at the surface of the small bowel. These lesions look like fibroids; biopsy was performed.

In addition, biopsy was carried out on multiple flat millimeter lesions located at the stomach surface. Moreover, no hepatic or peritoneal metastasis was found, as well as no lymphatic metastasis. Excision of the tumor was carried out with extemporaneous examination with negative surgical margins.

We proceeded a surgical resection of a part of the small intestine, in which exophytic nodules were located at. Histopathological examination revealed a stromal tumor in the stomach, small intestine, and duodenum. The postoperative course was simple with a dietary recovery at day 5. The patient was discharged 7 days postoperatively.

## 3. Discussion

Neurofibromatosis type I is also known as Von Recklinghausen disease after the German researcher, Friedrich Daniel von Recklinghausen, first described it in 1881 as a genetic neurodevelopmental disease, characterized by multisystemic disorders, including an increased risk of cognitive impairment ranging between 50 and 70%, and susceptibility to cancer [1]. Considered one of the most common genetic diseases, it has an estimated incidence of 1/3000 and a prevalence of 1/5000. Almost half of the people suffering from this disease were affected because of novo mutation [2]. Besides gastrointestinal manifestations and neuroendocrine tumors in patients with NF1, they share similarities with other conditions such as multiple endocrine neoplasia type 2B (MEN2B), Cowden syndrome (PTEN mutation), ganglioneuromas (GNs), and Proteus syndrome (PT); usually, those conditions share a genetic background of somatic mosaicisms implicating the PI3K-AKT-mTOR pathway activation which is now amenable to chemotherapy strategies [3].

The diagnostic criteria of neurofibromatosis type I were established in 1988, and it is confirmed when at least two of the following signs are found [4] (Table 1).

There is no accurate treatment to cure this disease. Recklinghausen disease occurs at middle age, usually much later than skin lesions, and can be divided into four entities [5]: (a) lesions of the intrinsic digestive nervous system and its supporting tissues, (b) stromal tumors, (c) duodenal neuroendocrine tumors or of the periampullary region, and (d) various tumors that do not belong to any of the previous tumors. It is noteworthy that the digestive system is affected by this disease in 12 to 60% of the cases.

As they are located next to the concerned digestive, the liver and the pancreas can also be affected. The incidence of stromal tumors of the digestive tract during Recklinghausen disease was approximatively up to 35% in the autopsy cases and only 5% in the clinical cases. Therefore, these tumors that can be either benign or malignant have a certain latency [6]. In case the patient suffers from multiple stromal tumors, it is important to look for Recklinghausen disease in both the patient and his family even in the absence of this disease's stigma. When dealing with Recklinghausen disease, one should always keep in mind the risk of a malignant tumor since it is four times higher in this disease than in the general population [7, 8]. It has been shown in the literature that histological diagnosis is not enough to find out if the tumor is malign or benign, leading to prognostic uncertainties. However, if the mitotic index is greater than five mitoses for 50 high-magnification fields and the size is greater than 5 cm, it is very much likely a malignant tumor [6]. The diagnostic circumstances of gastrointestinal stromal tumors are variable, including adventitious discovery, pain, mass syndrome, anemia, hem peritoneum, and especially gastrointestinal hemorrhage, which is the most frequent symptom.

Surgery remains the most used treatment for this disease. The only limitation facing this treatment is the multiple localizations of the gastrointestinal tumors, constraining the excision to symptomatic or complicated localizations [9]. Even patients with incomplete features to justify a diagnosis for NF1 still share common needs about the surgical management of some rare forms of tumors [10].

In all stages combined, survival after resection of malignant gastrointestinal stromal tumors is 35% at 5 years [11]. For an isolated primary tumor, survival is 50-56% at 5 years and 35-43% at 10 years with a more favorable prognosis for the stomach than for the small intestine [11, 12]. Surgery is the only effective treatment for an isolated tumor, while chemotherapy is deemed ineffective in the case of metastases [4, 13].

## 4. Conclusion

In cases with multiple stromal tumors, it is important that physicians and other caretakers be more aware of the potential association with Recklinghausen disease even in the absence of this disease's stigma knowing that they are at higher risk of a malignancy, since it is four times higher in this disease than in the general population.

## Figures and Tables

**Table 1:** Clinical diagnostic criteria for neurofibromatosis type I.

Diagnostic criteria of NF1
Two of the following signs
Six colored spots or more, measuring at least 5 mm before puberty and 15 mm after puberty
Lentigines in the axillary or inguinal region
Optic nerve glioma
Two neurofibromas of any type or plexiform fibroid
Two or more s
Bone abnormalities such as a dysplastic sphenoid bone or very fine long bone cortex
First-degree relative suffering from this disease
